# The Effect of *pstS* and *phoB* on Quorum Sensing and Swarming Motility in *Pseudomonas aeruginosa*

**DOI:** 10.1371/journal.pone.0074444

**Published:** 2013-09-04

**Authors:** Inna Blus-Kadosh, Anat Zilka, Gal Yerushalmi, Ehud Banin

**Affiliations:** 1 The Institute for Nanotechnology and Advanced Materials, Bar-Ilan University, Ramat Gan, Israel; 2 The Mina and Everard Goodman Faculty of Life Sciences, Bar-Ilan University, Ramat Gan, Israel; University of Oklahoma Health Sciences Center, United States of America

## Abstract

*Pseudomonas aeruginosa* is an opportunistic pathogen that can cause a wide range of infections and inflammations in a variety of hosts, such as chronic biofilm associated lung infections in Cystic Fibrosis patients. Phosphate, an essential nutrient, has been recognized as an important signal that affects virulence in *P. aeruginosa*. In the current study we examined the connection between phosphate regulation and surface motility in *P. aeruginosa*. We focused on two important genes, *pstS*, which is involved in phosphate uptake, and *phoB*, a central regulator that responds to phosphate starvation. We found that a mutant lacking *pstS* is constantly starved for phosphate and has a hyper swarming phenotype. Phosphate starvation also induced swarming in the wild type. The *phoB* mutant, on the other hand, did not express phosphate starvation even when phosphate was limited and showed no swarming. A double mutant lacking both genes (*pstS* and *phoB*) showed a similar phenotype to the *phoB* mutant (i.e. no swarming). This highlights the role of *phoB* in controlling swarming motility under phosphate-depleted conditions. Finally, we were able to demonstrate that PhoB controls swarming by up-regulating the Rhl quorum sensing system in *P. aeruginosa*, which resulted in hyper production of rhamonlipids: biosurfactants that are known to induce swarming motility.

## Introduction

*Pseudomonas aeruginosa* is a versatile bacterium that can adapt to a variety of niches and grows well in soil, water, plants and animals [1]. *P. aeruginosa* is also an opportunistic pathogen that can cause a variety of infections such as chronic infection of the lungs in Cystic Fibrosis (CF) patients [2], urinary tract infections resulting from catheterization [3], burn wounds [4] and Keratitis [5]. An important aspect of the *P. aeruginosa* “life style” is its ability to switch from a planktonic to a sessile mode of growth, depending on nutrient availability and its surrounding bacterial community. When nutrient availability is scarce, *P. aeruginosa* uses swarming motility, a coordinated movement on a surface, using flagella, biosurfactants [6] and Type-IV pili [7], in order to find an optimal niche [8]. In this mode of growth, planktonic cells differentiate to elongated and hyper-flagellated cells that spread across the surface. In order to facilitate the movement, *P. aeruginosa* secretes rhamnolipids [6], glycolipids that act as a biosurfactant and are produced by the *rhlAB* operon [9]. Rhamnolipid production is regulated by Quorum Sensing (QS) [10]–[12], a social behavior in which bacteria sense and respond to its surrounding population by producing and receiving signal molecules. There are three known QS systems in *P. aeruginosa* – Las and RhI, each consisting of an auto-inducer molecule from the acyl-homoserine lactone (AHL) family (synthesized by the LasI and RhlI enzymes) and a response regulator (LasR and RhlR) [13], and PQS (*Pseudomonas* Quinolone Signal), a secondary metabolite that functions as a QS molecule [14]. In addition to rhamnolipid production, QS in *P. aeruginosa* affects many bacterial processes, such as virulence [15] and biofilm formation [16]. While searching for irregular swarming phenotypes using a transposon mutant library, we came upon a hyper-swarming mutant. The mutated gene was mapped to *pstS*, a key component in the phosphate specific transport system (Pst) [17]. Phosphate is an essential nutrient, used in the assembly of ATP, LPS, nucleic acids and other cell components. *P. aeruginosa* has two phosphate uptake systems – Pit, a low-affinity, constitutively operating channel [18], and Pst – a high-affinity ABC transporter. The Pst system is encoded by the pst operon, containing the genes *pstA, pstB, pstC, pstS and phoU. pstA, pstB* and *pstC* encode the transporter proteins, while *pstS* encodes a periplasmic phosphate-binding protein that transfers the phosphate to the bacterial cytoplasm through the transporter. The system is regulated by PhoB/R – a two-component system activated by phosphate depletion [19]. When phosphate levels in the bacteria become low, PhoR phosphorylates PhoB, which in turn acts as a transcription factor and activates genes that have the consensus sequence ‘Pho Box’ in their promoters, such as the *pst* operon. In recent years, studies concerning the Pst system have uncovered a connection between phosphate depletion and virulence – over-expression of *PstS* has been found in ampicilin-resistant *Streptococcus pneumoniae* [20], a possible correlation between phosphate depletion and *P. aeruginosa* in a gut mouse model has also been suggested [21], and it has been shown that deletion of PhoB affects swarming [22]. Furthermore, the Pst system has been connected to virulence in many bacteria [23]. For example, in clinical strains of *P. aeruginosa*, *PstS* forms extracellular appendages that increase the strain’s virulence in a mouse model [24]. In this study, we establish the intricate connection between swarming, QS and phosphate availability in order to better understand the molecular mechanisms that regulate surface motility in *P. aeruginosa*. We show that in the absence of phosphate, PhoB up-regulates *rhlR* expression, which results in rhamnolipid production that promotes hyper swarming.

## Materials and Methods

### Bacterial Strains, Plasmids, and Media

The bacterial strains and plasmids used in this study are shown in Table 1. For Alkaline Phosphatase and Real-time assays, strains were grown on M9 minimal medium (20 mM NH4Cl; 12 mM Na2HPO4; 22 mM KH2PO4; 8.6 mM NaCl; 1 mM MgSO4; 1 mM CaCl2; 11 mM Dextrose) supplemented with 50 µM FeCl3. For swarming assays, we used M9 minimal medium or M9 depleted of phosphate (20 mM NH4Cl; 8.6 mM NaCl; 1 mM MgSO4; 1 mM CaCl2; 11 mM Dextrose), both supplemented with 0.5% Casamino acids, 50 µM FeCl3 and solidified with 0.5% Bacto Agar (Difco). For deletions, Luria-Bertani broth (LB, Difco), No Salt LB (NSLB, 0.5% Tryptone, 0.5% yeast extract), Vogel Bonner Minimal Medium (VBMM) and Psuedomonas Isolation Agar (PIA, Difco) were used. All strains were grown at 37°C with shaking, unless specified otherwise. Antibiotic concentrations used in this study were 300 µg/ml or 150 µg/ml carbenicillin for *P. aeruginosa*, 100 µg/ml ampicillin for *E. coli*, 100 µg/ml gentamicin for *P. aeruginosa* and 20 µg/ml for *E. coli*.

**Table 1:** Strains used in this study.

Strain or plasmid	Description	Source or reference
***P. aeruginosa strains***		
PAO1	Wild type	[36]
Δ*pstS*	PAO1 with an unmarked deletion of *pstS*	This study
Δ*phoB*	PAO1 with an unmarked deletion of *phoB*	This study
Δ*pstS*Δ*phoB*	PAO1 with an unmarked deletion of *pstS* and *phoB*	This study
Δ*rhlA*	PAO1 with an unmarked deletion of *rhlA*	This study
Δ*rhlR*	PAO1 with an unmarked deletion of *rhlR*	This study
Δ*pstS*Δ*rhlA*	PAO1 with an unmarked deletion of *pstS* and *rhlA*	This study
Δ*pstS*Δ*rhlR*	PAO1 with an unmarked deletion of *pstS* and *rhlR*	This study
***E. coli*** ** strains**		
DH5α	F′/*endA1 hsdR17 supE44 thi-1 recA1 gyrA relA1* Δ*(lac*ZYA*-argF)* U169*deoR (Φ80 dlac*Z-M15 *recA1)*	[37]
S17.1 (λpir)	*recA* derivative of *E. coli* 294 (F- *thi pro hsdR*) carrying a modifiedderivative of IncPα plasmid pRP4 (Aps Tcs Kms) integrated in thechromosome, Tpr; lysogenized with bacteriophage λpir	Y. Irie and M. R. Parsek
***Plasmids***		
pUCP18Ap	A broad-host range cloning vector. CbR/AmpR	[38]
DB3.1 pEX18GmGW	pEX18Gm containing the Gateway (GW) destination cloning site. GmR	Nan Fulcher and Matthew Wolfgang
*pphoB*	pUCP18Ap containing the *phoB* gene for complementation	This study
*ppstS*	pUCP18Ap containing the *pstS* gene for complementation	This study
*prhlA*	pUCP18Ap containing the *rhlA* gene for complementation	This study
*prhlR*	pUCP18Ap containing the *rhlR* gene for complementation	This study
pECP61.5	Contains a *rhlA-lacZ* fusion, used for C4-HSL detection	[11]

AmpR -Ampicilin resistance for *E. coli* CbR – Carbenicillin resistance for *P. aeruginosa*. GmR – Gentamicin resistance.

### Construction of Strains and Plasmids

Deletion mutants of *pstS*, *phoB*, *pstS*+*phoB*, rhlA and rhlR were constructed as previously described [25]. Overlap extension PCR using the primers specified in Table S1 was used in order to generate a fragment containing the upstream and downstream regions of each gene. Each fragment was cloned into the allelic exchange vector DB3.1 pEX18GmGW [26] using BP-Clonase (Invitrogen). Each deletion was introduced to PAO1 or Δ*pstS* using bi-parental mating [27]. Deletions were generated using a standard method for two-step allelic exchange as described by Schweizer and Hoang [28] and were confirmed by PCR.

### Swarming Motility Assay

Strains were grown overnight in M9 medium supplemented with Casamino acids, FeCl3, and carbenicillin if necessary. Afterwards, the bacteria were diluted 1∶10 into fresh medium and grown for an additional three hours in order to reach the logarithmic growth phase. 2.5 µl from each culture was plated in the middle of a swarming plate, which contained M9 medium or M9 depleted of phosphate, supplemented with 0.5% Casamino acids, 50 µM FeCl3 and carbenicillin if necessary, and solidified with 0.5% Bacto Agar. Plates were incubated at 37°C for 24 hours.

### Alkaline Phosphatase Assay

Alkaline Phosphatase (AP) is a protein whose expression is enhanced under phosphate starvation [29]. We utilized AP activity in order to assay a strain’s phosphate starvation levels. AP activity was measured by sampling strains grown in a liquid culture or on swarming plates. To measure AP from a liquid culture, strains were grown overnight in M9 medium supplemented with FeCl3, and carbenicillin if necessary. Afterwards, bacteria were diluted to an O.D595nm of 0.04 into 50 ml of fresh M9 medium, containing 50 µM FeCl3 and grown for an additional 10 hours. Then, 6 ml from each strain were taken and centrifuged for 10 minutes at 2,200 g (Centrifuge 5418; eppendorf (. The pellet was re-suspended with 20 µl of Chloroform. After 15 minutes of incubation at room temperature, 20 µl of 0.01 M Tris-HCl (pH = 8) were added to each sample, and the samples were centrifuged for 20 minutes at 6,000 g. Following centrifugation, 30 µl from each sample’s supernatant were added to a 96-well plate, containing the reaction buffer (5 µl of 0.5 mM MgCl2 and 10 µl of 1 M Tris (pH = 9.5)). 5 µl of 500 mM p-Nitrophenyl Phosphate (PNPP, NEB) were added to each well and the reaction was read at 405 nm in an ELISA plate reader )Synergy™ 2 Multi-Detection Microplate Reader; Biotech). Results were normalized to each samples’ total protein concentration using Bradford assay (Thermo).

When measuring AP activity from swarming plates, bacteria grown for 24 hours on swarming plates were scraped off and suspended in 2 ml of M9, then submitted to the same procedure as the samples taken from liquid culture.

### RNA Extraction, RT-PCR and Real-Time PCR Analysis

Strains were grown overnight in M9 medium supplemented with FeCl3. Then, bacteria were diluted to an O.D595nm of 0.04 into 50 ml of fresh M9 medium, containing FeCl3 and grown for an additional 48 hours. Afterwards, 2 ml from each culture was incubated with 4 ml of RNAprotect Bacteria Reagent (Qiagen). RNA extraction was performed using RNeasy Mini Kit (Qiagen). cDNA was synthesized from 500 ng of RNA using GoScript™ Reverse Transcription System (Promega). Real-time PCR was performed using CFX-96 Touch Real-Time PCR detection system (Bio-Rad). Results were normalized to the expression of PA1769 [30].

### C4–HSL Quantification

The level of C4-HSL was measured using a bio-reporter strain as previously reported [31]. Briefly, strains were grown overnight in M9 medium supplemented with FeCl3. Then, bacteria were diluted to an O.D595nm of 0.04 into 50 ml of fresh M9 medium, supplemented with FeCl3 and grown for an additional 48 hours. Afterwards, 5 ml from each culture was extracted twice with an equal volume of ethyl acetate containing 0.1% glacial acetic acid, as previously described [32]. Ethyl acetate was evaporated using nitrogen gas. Overnight culture of DH5α harboring pECP61.5 was diluted into an O.D595nm of 0.1 into modified A medium [33] with ampicillin and 1 mM IPTG. 500 µl of the diluted bacteria was added to each sample and grown at 30°C for 5.5 hours. Cells were than lysed with chloroform and the β-galactosidase activity was measured using Tropix-Galacton kit according to the manufacturer guidelines (Applied Biosystems).

### Statistical Analysis

Statistical analysis was carried out using unpaired t-test and Turkey’s Post Hoc test. P<0.05 was considered as significant.

## Results and Discussion

### Phosphate Starvation Promotes *phoB*-mediated Hyper Swarming

**Figure 1:**
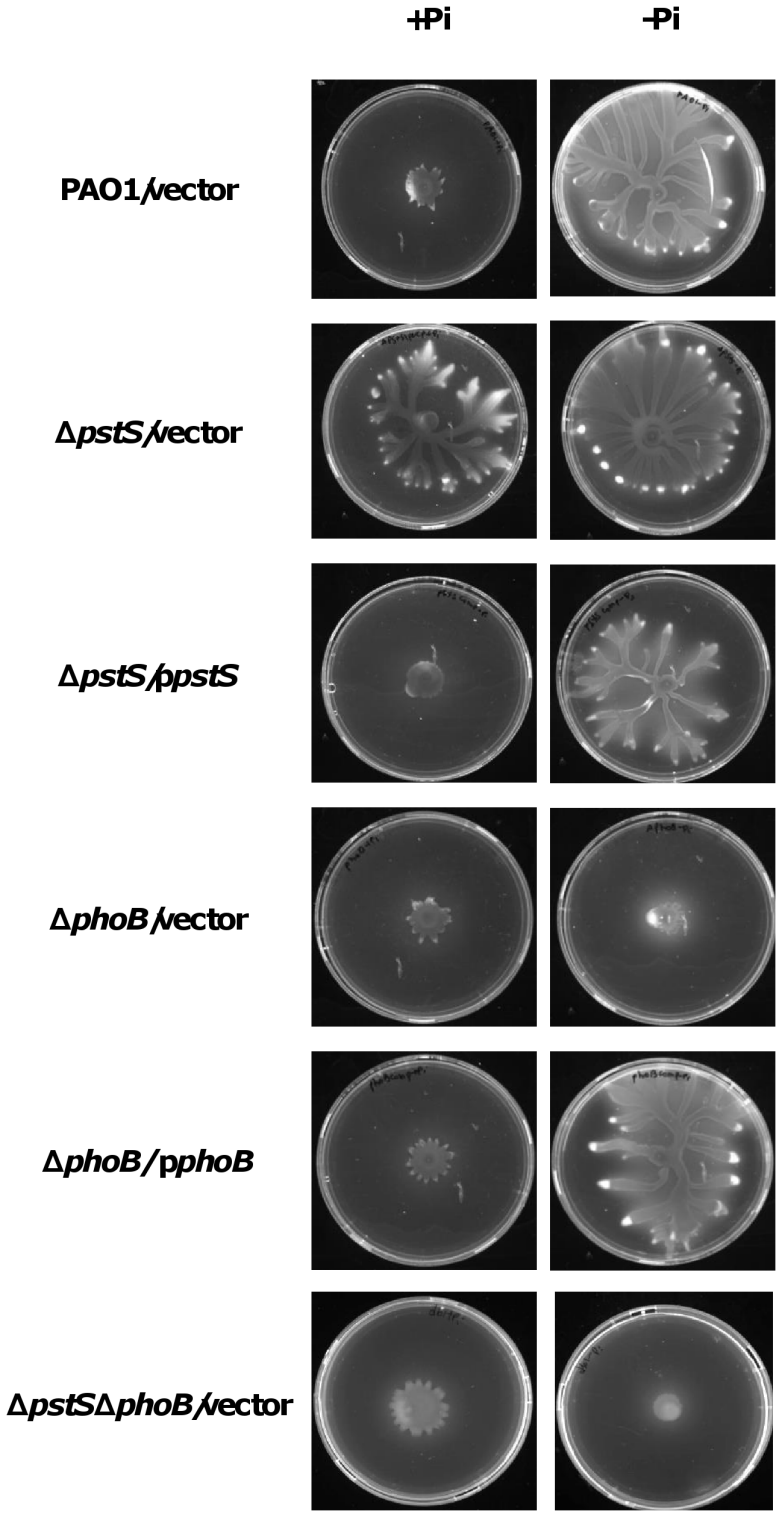
Influence of *pstS* and *phoB* deletion on swarming motility. PAO1, Δ*pstS*, Δ*phoB* and Δ*pstS*Δ*phoB* carrying an empty vector (pUCP18Ap) or a complementation plasmid (p*pstS* and p*phoB*) were grown for 24 hours at 37°C on swarming plates containing M9 (20 mM) or phosphate-depleted M9 (0.2 mM) as described in Materials and Methods.

While scanning a transposon mutant library for irregular swarming phenotypes, we found that transposon insertion in the *pstS* gene promoted hyper-swarming. In order to ensure that the phenotype is indeed caused by the disruption of the gene, we generated a clean deletion of *pstS* in PAO1. The mutant showed a hyper-swarming phenotype both in standard M9 minimal medium representing phosphate-repleted conditions (20 mM Pi) and in M9 medium with phosphate depleted conditions (0.2 mM Pi). The wild type, on the other hand, showed swarming ability only under phosphate-depleted conditions (Fig. 1). Complementation of the Δ*pstS* strain with a plasmid encoding *pstS* restored the wild type phenotype.

Next, we generated a clean deletion of *phoB* in PAO1, in order to see how deletion of a protein with a regulatory function in the phosphate uptake system affects the bacteria’s swarming pattern. We saw that deletion of *phoB* caused the bacteria to lose all swarming ability, even under phosphate depleted conditions. A similar result was demonstrated previously by Bains *et al.* [22], who showed that a *phoB* mutant loses its ability to swarm when grown on a BM2 medium with low (0.2 mM) phosphate concentration. Complementation of our Δ*phoB* strain with a plasmid encoding *phoB* restored the wild type phenotype (Fig. 1). We then wanted to see which one of the two genes – *pstS* or *phoB* – is responsible for controlling the swarming phenotype under phosphate depleted conditions. To do so, we generated a double mutant, lacking both *pstS* and *phoB* in PAO1. We assumed that if PstS controlled swarming in the presence or lack of phosphate, the double mutant would show the hyper-swarming phenotype presented by the *pstS* mutant, and if PhoB controlled swarming under the tested conditions, the double mutant would act like the *phoB* mutant and show a non-swarming phenotype. Plating the double mutant on swarming plates containing M9 or M9 with low levels of phosphate, generated a non-swarming phenotype under both conditions (Fig. 1), which led us to conclude that PhoB is responsible for controlling *P. aeruginosa*’s swarming patterns in the presence or lack of phosphate. When we complemented the double mutant with a plasmid containing the *pstS* gene, we received the phenotype presented by *phoB*, and when we complemented the double mutant with a plasmid containing the *phoB* gene (p*phoB*), we received the phenotype presented by *pstS* (Fig. S1A). From these experiments we can conclude that while both *pstS* and *phoB* have impact on swarming motility on media containing low levels of phosphate, PhoB is the protein that regulates swarming under the specified conditions.

### The *pstS* Mutant is Constantly Starved for Phosphate, while the *phoB* Mutant cannot sense the Bacteria’s Phosphate Levels

**Figure 2:**
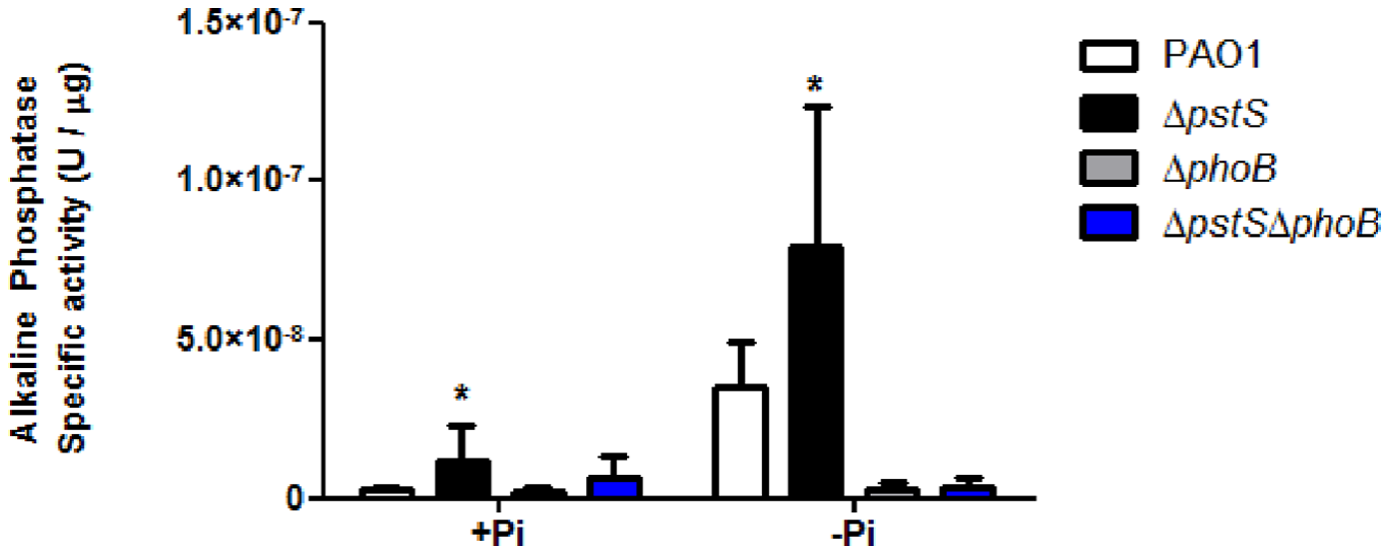
The effect of *pstS* and *phoB* deletion on phosphate starvation in *P.aeruginosa*. PAO1, Δ*pstS*, Δ*phoB* and Δ*pstS*Δ*phoB* were grown for 24 hours at 37°C on swarming plates containing M9 or phosphate-depleted M9. After 24 hours, bacteria were scraped off the plates and Alkaline Phosphatase activity was measured using p-Nitrophenyl Phosphate as described in Materials and Methods. Results were normalized to each samples’ total protein concentration using Bradford assay. Results shown represent mean+standard deviation of six different experiments. Each experiment was performed in triplicate. Asterisks represent the significant rise in AP activity compared to the WT (p<0.05, student’s t-test).

The results from the swarming experiments led us to hypothesize that deletion of *pstS* causes *P. aeruginosa* to be constantly starved for phosphate, whereas deletion of *phoB* causes the bacteria to lose its ability to sense its intra-cellular phosphate levels. Because PhoB is activated under low phosphate levels, we assumed that deletion of *phoB* causes the bacteria to act as if they were in a constant state of phosphate saturation. To test this hypothesis, we plated PAO1, Δ*pstS*, Δ*phoB* and Δ*pstS*Δ*phoB* on either phosphate depleted or repleted swarming plates in order to measure the Alkaline Phosphatase (AP) activity in each strain, as described in Materials and Methods. AP levels increase when intra-cellular phosphate levels are low, thus, AP levels point to the degree of phosphate starvation in bacteria. PAO1 was used as a reference – as expected, when grown on standard M9 plates, AP levels were low, and when grown on phosphate-depleted M9, AP levels increased dramatically (p<0.05) (Fig. 2). AP levels in the *pstS* mutant were higher than in PAO1 in both media (p<0.05), and AP levels in Δ*phoB* and Δ*pstS*Δ*phoB* were similar to those of PAO1 grown on standard M9 plates, but did not increase under phosphate depleted conditions (Fig 2). Complementation of the deletion strains, with plasmids encoding *pstS* or *phoB,* reverted the phenotypes (Fig. S1B). The results suggest that absence of both *pstS* and *phoB* disrupts *P. aeruginosa*’s ability to respond to its intra-cellular phosphate levels, but in different ways. Deletion of *pstS*, the gene that encodes the phosphate-carrying protein, causes the bacteria to lose its ability to acquire phosphate through the Pst system, which puts the bacteria in a state of constant phosphate starvation. On the other hand, deletion of *phoB* causes *P. aeruginosa* to lose the regulatory part of the phosphate uptake system. Since PhoB/R are activated only when phosphate levels are low, bacteria with a *phoB* deletion will lose their ability to sense when there is a decrease in intra-cellular phosphate levels and therefore will always have a false sense of being in a phosphate saturated environment, hence the lack of swarming and the low AP levels in Δ*phoB* and Δ*pstS*Δ*phoB* grown in media containing low levels of phosphate.

### *rhlR* and *rhlA* are Essential for Swarming Under Phosphate-deplete Conditions

**Figure 3:**
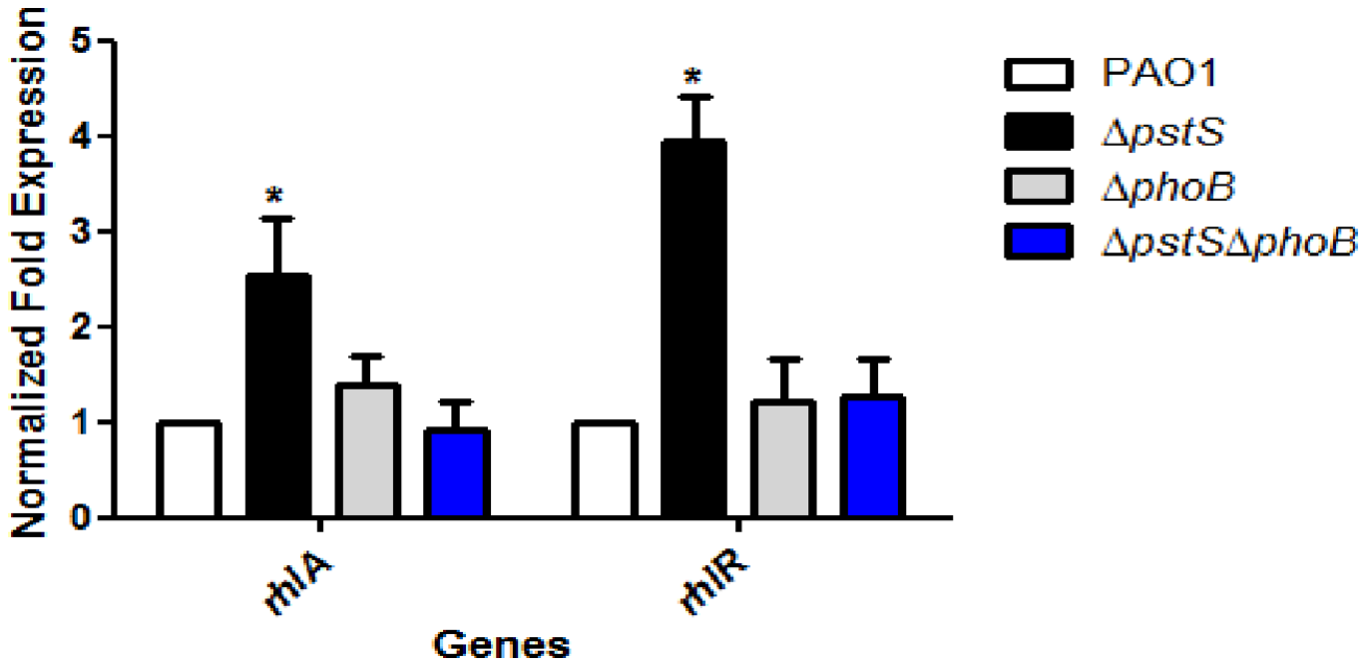
*pstS* knockout results in elevated *rhlA* and *rhlR* expression. PAO1, Δ*pstS*, Δ*phoB* and Δ*pstS*Δ*phoB* were grown for 48 hours at 37°C with shaking in M9 medium_._ Levels of *rhlA* and *rhlR* were measured by Real-Time PCR as described in Materials and Methods. Values were normalized to the expression of PA1769. The results shown are relative to PAO1 and represent mean+standard deviation of nine different experiments. Each experiment was performed in triplicate. Asterisks represent the significant elevation in *rhlA* and *rhlR* transcription compared to the WT (p<0.05, student’s t-test).

**Figure 4:**
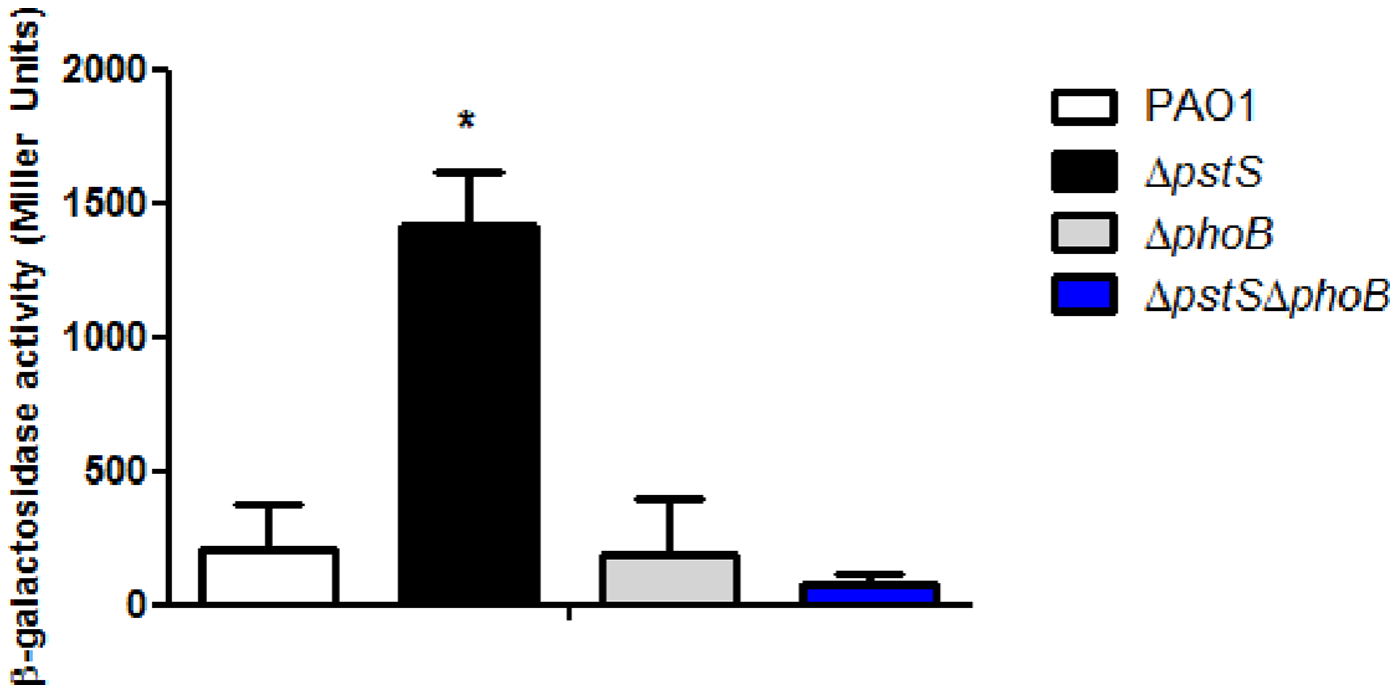
Phosphate starvation promotes C4-HSL production. PAO1, Δ*pstS*, Δ*phoB* and Δ*pstS*Δ*phoB* were grown for 48 hours at 37°C with shaking in M9 medium_._ C4-HSL in each strain was extracted and measured as described in Materials and Methods. β-galactosidade activity (Miller Units) represent C4-HSL levels. Results represent mean+standard deviation of three different experiments. Each experiment was performed in triplicate. Asterisks represent the significant elevation in C4-HSL production in Δ*pstS* as opposed to all other strains (p<0.05, Turkey’s post hoc test).

After determining how deletion of *pstS* and *phoB* affects *P. aeruginosa*’s ability to sense phosphate, we were interested to study the molecular pathway that leads from the bacteria’s reaction to phosphate depletion to swarming motility. It was previously shown that the *rhlR* promoter contains a Pho Box, that *rhlR* transcription can be activated under phosphate-limiting conditions [34] and that RhlR controls rhamnolipid production [10]. Therefore, we wanted to see if up-regulation of *rhlR*, and in turn, up-regulation of *rhlA* and increase in rhamnolipid production, is the cause for hyper-swarming under phosphate-depleted conditions. In order to do so, we measured the transcription levels of *rhlR* and *rhlA* in PAO1, Δ*pstS*, Δ*phoB* and Δ*pstS*Δ*phoB* grown in M9 medium, using Real-Time PCR. Results were normalized to those of PAO1, the reference strain (Fig. 3). Only in Δ*pstS* did we see a significant rise in transcription levels of *rhlA* and *rhlR* (2.5 and 3.9 fold elevation in gene transcription as opposed to the WT, respectively, p<0.05). Because Δ*pstS* remains constantly starved for phosphate, PhoB is activated in this strain, which strengthens our hypothesis that PhoB is indeed responsible for setting in motion the cascade that ultimately results in swarming in the absence of phosphate. This result is further supported by microarray studies done by Bains et al., showing that *rhlA* and *rhlR* transcription increases under minimal phosphate conditions in *P. aeruginosa* [22]. To further corroborate this data, we measured the C4-HSL levels in each of the strains (Fig. 4). Coinciding with the RT-PCR results, C4-HSL levels in Δ*pstS* were significantly higher than those of the other strains (p<0.05), which shows that the response to phosphate starvation involves all factors of the Rhl system.

**Figure 5:**
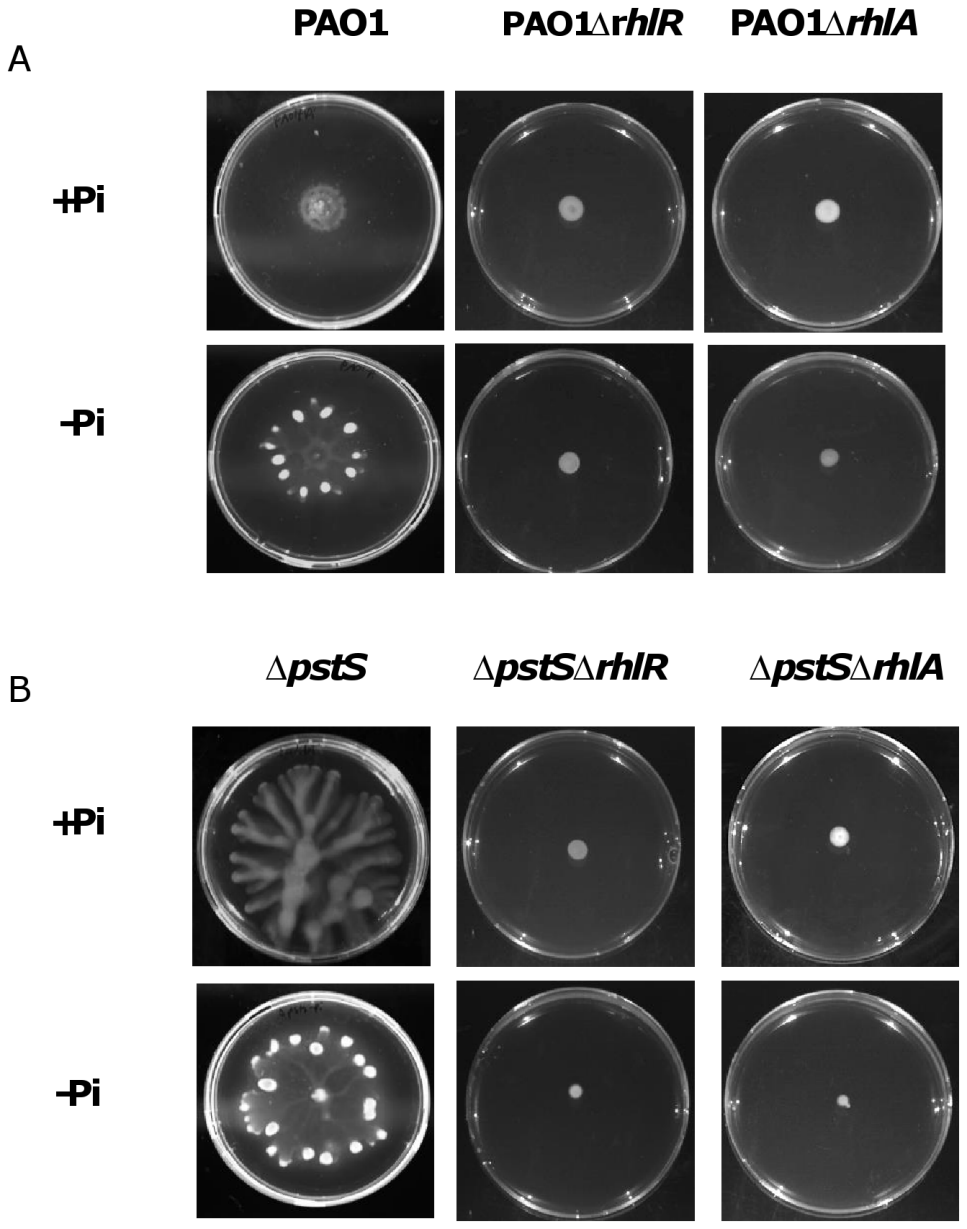
*rhlA* and *rhlR* deletion cause loss of swarming motility. A. PAO1, PAO1Δ*rhlA* and PAO1Δ*rhlR* were grown for 24 hours at 37°C on swarming plates containing M9 or phosphate-depleted M9. B. Δ*pstS*, Δ*pstS*Δ*rhlA* and Δ*pstS*Δ*rhlR* were grown for 24 hours at 37°C on swarming plates containing M9 or phosphate-depleted M9.

Next, in order to further prove that *rhlR* and *rhlA* are responsible for hyper-swarming when phosphate level is low, we generated clean deletions of *rhlR* and *rhlA* in PAO1 and in Δ*pstS*, and plated them on swarming plates containing M9 with either low or high levels of phosphate. The results (Fig. 5) clearly show that deletion of each of these genes abolished the swarming completely in both strains and in both media, which proves that these genes are crucial for *phoB*-mediated swarming when phosphate is low. Complementation of *rhlA* and *rhlR* restored the phenotypes of both PAO1 and Δ*pstS* (data not shown).

**Figure 6:**
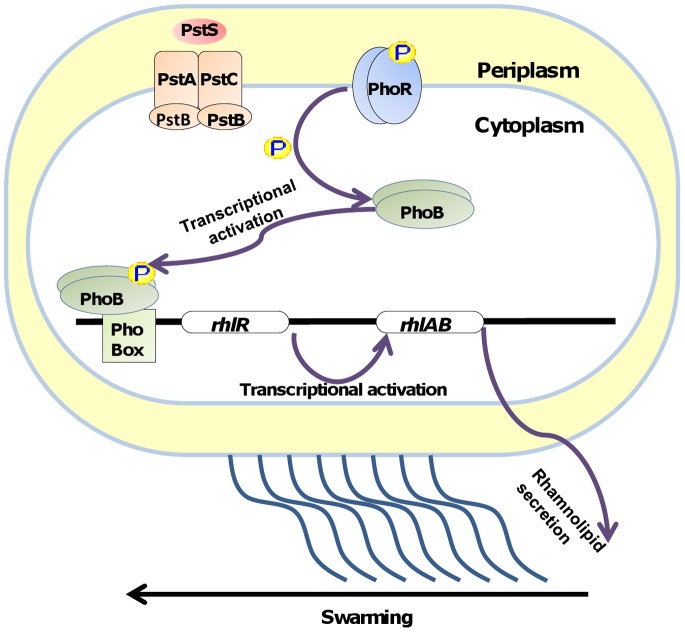
Model of the regulatory pathway leading to swarming under phosphate depletion.

All of these results combined piece together the molecular mechanism that is initiated under phosphate-deplete conditions (Fig. 6) - PhoB is activated upon phosphorylation by PhoR, acts as a transcription factor and binds to promoters containing a Pho Box motif. One of these promoters is the promoter for *rhlR*. RhlR undergoes activation, and activates transcription of the *rhlAB* operon, causing rhamnolipid production, which results in swarming. Interestingly, we have previously shown in *P. aeruginosa* that iron depletion can also cause increased motility by QS-mediated rhamnolipid production [35]. The current study further links between environmental cues and QS, and sheds light on the complicated regulatory network in *P. aeruginosa* that makes this pathogen so versatile and highly adjustable to a variety of niches.

## Supporting Information

Figure S1 **Complementation of** ***pstS*** **and** ***phoB*** **restores the wild type swarming phenotype.**
(TIF)

Table S1 Primers used in this study.
(DOCX)
