# Supplementary figures and images for: The Effect of pstS and phoB on Quorum Sensing and Swarming Motility in Pseudomonas aeruginosa
Source: PLoS One. 2013 Sep 4;8(9):e74444. doi: 10.1371/journal.pone.0074444 (PMC3762822; doi:10.1371/journal.pone.0074444)

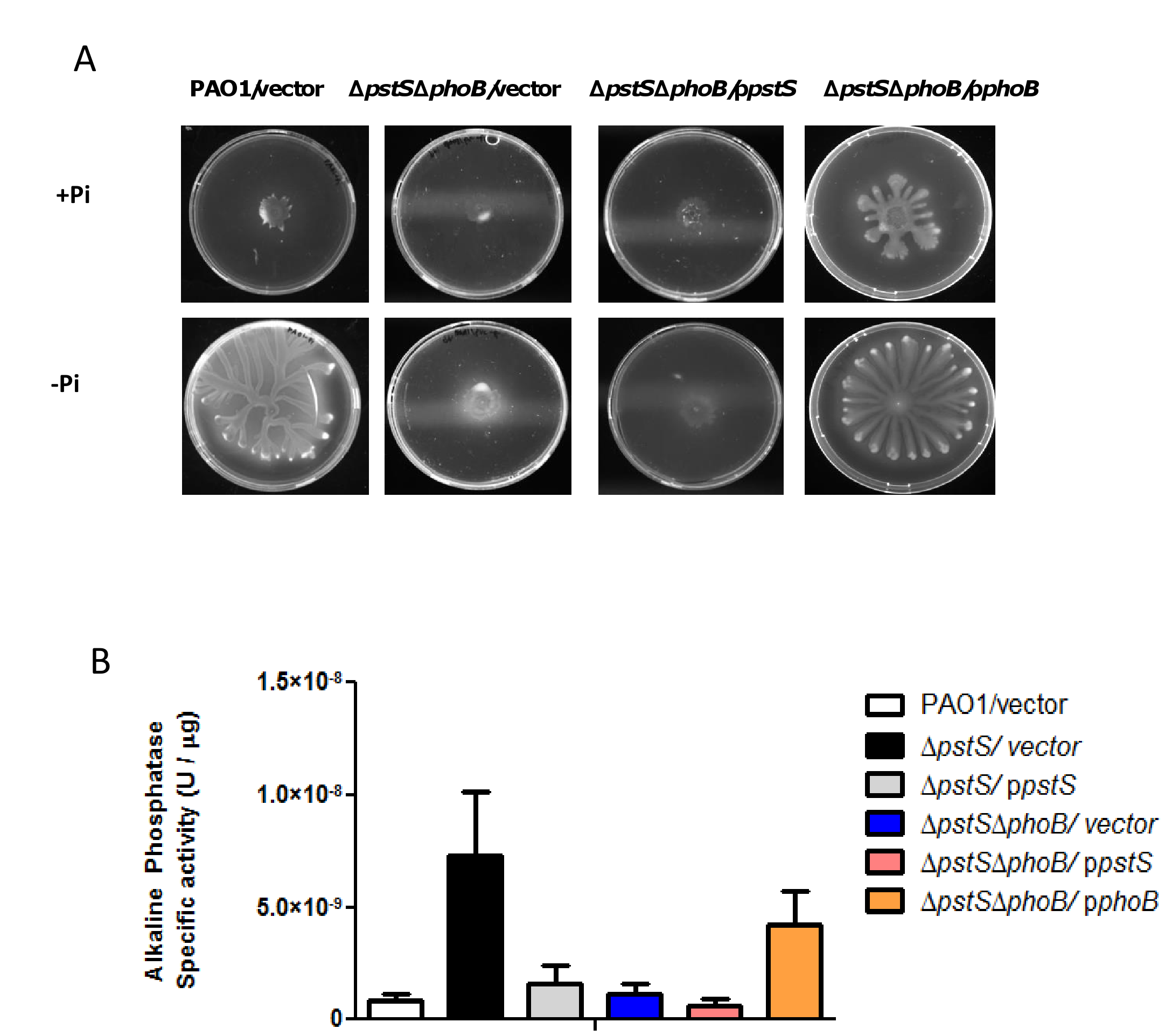

Supplement: Figure S1 — Complementation of pstS and phoB restores the wild type swarming phenotype. (TIF) [file pone.0074444.s001.tif]
