# Supplementary material for: The Effect of pstS and phoB on Quorum Sensing and Swarming Motility in Pseudomonas aeruginosa
Source: PLoS One. 2013 Sep 4;8(9):e74444. doi: 10.1371/journal.pone.0074444 (PMC3762822; doi:10.1371/journal.pone.0074444)
Supplement: Table S1 — Primers used in this study. (DOCX) [file pone.0074444.s002.docx]

Table S1. **Primers used in this study**

| **Primer** | **Sequence (5' to 3')** | **Used for** | **Underlined** |
| --- | --- | --- | --- |
| 1229 | GGGGACAAGTTTGTACAAAAAAGCAGGCTCAGATTGCCCTTCGCTGTCCTT | *phoB*  knockout |  |
| 1230 | GCGGGGTCAGCTCTTGGTGGCCACGGCGATCATCTC | *phoB*  knockout |  |
| 1231 | ACCAAGAGCTGACCCCGC | *phoB*  knockout |  |
| 1232 | GGGGACCACTTTGTACAAGAAAGCTGGGTACGTCCTGCGGGGTCTTCA | *phoB*  knockout |  |
| 1147 | AGAG**AAGCTT**AACCTGTTGAGCATAGCTC | *phoB*  complementation | *Hind*III site |
| 1148 | AGAG**GAATTC**TCAGCTCTTGGTGGAGAAAC | *phoB*  complementation | *EcoR*I site |
| PstSUpF01-GWB1 | GGGGACAAGTTTGTACAAAAAAGCAGGCTCACAATTGCCCTGGAAACTACC | *pstS*  knockout |  |
| PstSUpR01 | TACAGGCCCAGTTCCTTGATCGCCGGCCGCCATCAAACGCTT | *pstS*  knockout |  |
| PstSDownF01 | GGCGATCAAGGAACTGGG | *pstS*  knockout |  |
| PstSDownR01-GWB2 | GGGGACCACTTTGTACAAGAAAGCTGGGTACGACCAGCACGTACCAG | *pstS*  knockout |  |
| pstsF | AGAG**AAGCTT**CTATCCCAAAACCCCTGGTC | *pstS*  complementation | *Hind*III site |
| pstsR | AGAG**GAATTC**TCCGCTCAAGTCACTGGATT | *pstS*  complementation | *EcoR*I site |
| 1613 | GGGGACAAGTTTGTACAAAAAAGCAGGCTCAGACAATGCCGAACGGCTG | *rhlR*  knockout |  |
| 1614 | TCAGATGAGACCCAGCGCCCTCATTGCAGTAAGCCCTG | *rhlR*  knockout |  |
| 1615 | GCGCTGGGTCTCATCTGA | *rhlR*  knockout |  |
| 1616 | GGGGACCACTTTGTACAAGAAAGCTGGGTAAGACCACGTCCCAGCCCA | *rhlR*  knockout |  |
| 1617 | GGGGACAAGTTTGTACAAAAAAGCAGGCTCAGAGGCCTGCGAAGTGTCC | *rhlA*  knockout |  |
| 1618 | TCAGGCGTAGCCGATGGCCAGACTTTCGCGCCGCAT | *rhlA*  knockout |  |
| 1619 | GCCATCGGCTACGCCTGA | *rhlA*  knockout |  |
| 1620 | GGGGACCACTTTGTACAAGAAAGCTGGGTATCGTAGACCGGCTCGATC | *rhlA*  knockout |  |
| 1688 | AGAG**GAATTC**AAATCTGGCAGTTACCGT | *rhlA* complementation | *EcoR*I site |
| 1689 | AGAG**AAGCTT**TCTTCGCAGGTCAAGGGT | *rhlA* complementation | *Hind*III site |
| 1690 | AGAGGAATTCGCGCGCTTGACAGCGTCA | *rhlR* complementation | *EcoR*I site |
| 1691 | AGAGAAGCTTTCAGATGAGACCCAGCGC | *rhlR* complementation | *Hind*III site |
| 1557 | TGATCGATATCTTCGCCACCTT | RT-PCR of PA1769 |  |
| 1558 | GGGACTTGCCGACCGAATA | RT-PCR of PA1769 |  |
| 1559 | AACGAGACCGTCGGCAAATAC | RT-PCR of *rhllA* |  |
| 1560 | AGCACCTGGTCGATGTGAAAG | RT-PCR of *rhllA* |  |
| 1561 | GGGAAATCGCCATCATCCT | RT-PCR of *rhllR* |  |
| 1562 | GCGCGTCGAACTTCTTCTG | RT-PCR of *rhllR* |  |
